# A 1‐year follow‐up of the My Grief app for prolonged grief

**DOI:** 10.1002/jts.23181

**Published:** 2025-06-20

**Authors:** Maarten C. Eisma, Lara O. Schmitt, Rakel Eklund, Filip K. Arnberg, Paul A. Boelen, Josefin Sveen

**Affiliations:** ^1^ Department of Clinical Psychology and Experimental Psychopathology University of Groningen Groningen the Netherlands; ^2^ Department of Women's and Children's Health Uppsala University, Uppsala University Hospital Uppsala Sweden; ^3^ National Centre for Disaster Psychiatry, Department of Medical Sciences Uppsala University, Uppsala University Hospital Uppsala Sweden; ^4^ Department of Clinical Psychology Utrecht University Utrecht the Netherlands; ^5^ ARQ National Psychotrauma Centre Diemen the Netherlands; ^6^ Centre for Crisis Psychology University of Bergen Bergen Norway

## Abstract

Mobile health applications (apps) are increasingly used to reduce mental health problems. However, few effective apps are available for bereaved adults. Recently, a randomized controlled trial demonstrated the short‐term beneficial effects of access to the My Grief app in mitigating symptoms of prolonged grief and posttraumatic stress in bereaved parents. The present study examined the long‐term outcomes of app access and their predictors in a longitudinal survey of participants who had access to the My Grief app. We assessed symptoms of prolonged grief (PG‐13), posttraumatic stress (PCL‐5), and depressive symptoms (PHQ‐9) at 3‐, 6‐, and 12‐month follow‐up assessments. Potential predictors of symptom change included baseline symptom levels, sociodemographic and loss‐related variables, rumination (UGRS), negative grief‐related cognitions (GCQ‐SF), avoidance processes (DAAPGQ), and self‐reported app use reported at each follow‐up. Significant small‐to‐moderate reductions in prolonged grief, posttraumatic stress, and depressive symptoms were observed in people with app access at most follow‐ups, *ds* = 0.26–0.66. For each symptom type, more severe baseline symptoms significantly predicted larger symptom reductions, *B*s = 0.37–0.55. Lower baseline negative grief‐related cognitions significantly predicted larger 3‐month prolonged grief, *B* = −0.15, and posttraumatic stress symptom reductions, *B* = −0.23. Lower baseline anxious avoidance significantly predicted larger 3‐month depressive symptom reductions, *B* = −0.23. Self‐reported app use did not significantly predict symptom changes. Participants with access to the My Grief app experienced decreased symptom levels over a 1‐year period. Specific cognitive behavioral processes (avoidance, negative cognitions) appear to be implicated in the short‐term effects of app access.

Mobile health (mHealth) interventions are useful, feasible, acceptable, and efficacious for a wide range of mental health conditions, including bipolar disorder, alcohol abuse, and insomnia (e.g., Bush et al, 2019; Wang et al., 2018). Mobile applications (apps) can be applied for multiple purposes, including psychoeducation, illness self‐management, and symptom monitoring (Naslund et al., 2015). Apps offer potential advantages over traditional face‐to‐face and internet‐based interventions by providing easily accessible, low‐cost, anonymous, and immediate support (Olff, 2015). Despite these advantages, the proven effectiveness, and increasing scientific interest in online interventions for prolonged grief (e.g., Lenferink et al., 2023; Treml et al., 2021; Wagner et al., 2022; for a review, see Zuelke et al., 2021), no empirically supported mobile apps existed for bereaved adults until recently.

The My Grief app (in Swedish, *Min Sorg*), partly based on the well‐investigated PTSD Coach app (e.g., Hensler et al., 2022; Kuhn et al., 2017; for a review, see Bröcker et al., 2023) and including elements taken from cognitive behavior therapy (CBT) for prolonged grief (e.g., Boelen & Eisma, 2022), was recently developed to reduce prolonged grief in parents who have lost a child (Eklund et al., 2021). Though the evidence for PTSD Coach is mixed, with a recent meta‐analysis finding a small, nonsignificant effect on posttraumatic stress disorder (PTSD) symptoms (Bröcker et al., 2023), findings on the My Grief App appear promising. An open pilot trial among 13 bereaved parents demonstrated that 1‐month app access coincided with large reductions in prolonged grief symptoms (Eklund et al., 2022). Moreover, participants reported that the app was easy to use, normalized grief, and provided useful information and further support, and they stated they would recommend the app to other parents. These initial findings were extended in a randomized waitlist‐controlled trial among 248 bereaved parents, which demonstrated that 3‐month access to the My Grief app resulted in small, yet statistically significant, reductions in prolonged grief symptoms and posttraumatic stress symptoms (PTSS) but not depressive symptoms (Sveen et al., 2024). Moreover, participants who received app access endorsed its usability, feasibility, and acceptability and reported few negative experiences (Eklund et al., 2024; Sveen et al., 2024). Together, these results support the potential usefulness of the My Grief app as a complementary tool to improve intervention options for adults with prolonged grief.

Though the short‐term effects of the My Grief app are encouraging, it remains to be established whether symptom reductions are maintained over time, thus providing further information on the app's clinical value. Research on the long‐term outcomes of online grief interventions is rare. However, two prior uncontrolled follow‐up studies demonstrated that the effects of a therapist‐guided, internet‐based writing intervention on depressive symptoms, anxiety symptoms, prolonged grief symptoms, and PTSS were maintained at 1‐ and 1.5‐year follow‐ups (Wagner & Maercker, 2007; Kersting et al., 2013). The long‐term effects of unguided online grief interventions, such as the My Grief app, have not yet been investigated. The primary aim of the present study was, therefore, to clarify the long‐term outcomes of access to the My Grief app. We predicted that short‐term reductions in prolonged grief symptoms and PTSS following app access would be maintained over a 1‐year period. For exploratory purposes, we also analyzed the temporal changes in depressive symptoms in the same period.

Given the paucity of research on the topic, it is unclear which bereaved adults benefit most from app access. This is critical information, as it may help clarify to whom this intervention can best be offered. Prior work on factors predicting outcomes of grief‐focused psychotherapy showed that sociodemographic and loss‐related characteristics, symptom levels, and cognitive behavioral variables are related to treatment gains. For example, CBT for prolonged grief has been found to be more effective for individuals who have lower levels of educational attainment; have lost a partner or child; report higher baseline prolonged grief symptoms (Boelen et al., 2011); and experience lower levels of self‐blame, maladaptive avoidance (Bryant et al., 2017), and negative grief‐related beliefs (Skritskaya et al., 2020). Additionally, in a rare study of predictors of online therapist‐guided CBT, higher baseline levels of prolonged grief symptoms, self‐efficacy, and attachment anxiety were found to be related to larger treatment gains (Schmidt et al., 2022). Therefore, the secondary aim of this study was to expand this line of research in the context of an app for prolonged grief. Specifically, we explored the associations among baseline symptom levels, negative grief‐related cognitions, grief rumination, and avoidance behaviors and short‐ and long‐term changes in prolonged grief, posttraumatic stress, and depressive symptoms during 12‐month access to the My Grief app.

## METHOD

### Participants

Individuals were eligible for participation if they were parents who (a) experienced the death of a child 1–10 years previously, (b) reported elevated levels of prolonged grief (i.e., a score of 17 or higher on the Prolonged Grief Disorder–13 scale [PG‐13; Prigerson et al., 2009]), (c) mastered the Swedish language, and (d) had access to a smartphone. Participants with self‐reported current suicidal thoughts or psychosis, measured using single screening items, were excluded.

### Procedure

#### Study design

This study was part of a two‐armed parallel‐group randomized waitlist‐controlled trial (Clinicaltrials.gov, identifier: NCT04552717; Sveen et al., 2024). For this study, we analyzed online survey data from the treatment arm; all participants had access to the My Grief app for 12 months. For ethical reasons, the control group also received app access after 3 months, but they did not complete follow‐up surveys.

#### Recruitment, eligibility, and ethical approval

Participants were recruited from September 2021 to January 2022. The recruitment was divided into two phases. First, potential participants were identified using the Swedish Childhood Cancer Registry and the Cause of Death Registry to find children who had been diagnosed with malignancies and died between 2010 and 2020. Through the Swedish Population Registry, the parents of the children who died were identified, and an invitation letter for this study was mailed between September 2021 and November 2021. The second recruitment phase occurred in January 2022 and included advertisements for the study posted on the social media accounts and in the newsletters of two nonprofit organizations for bereaved parents in Sweden. Parents interested in participation completed an online screening questionnaire via a secured website (RedCap). Upon the confirmation of eligibility, participants were directed to an informed consent form and a baseline questionnaire. Participants randomized to the treatment arm were provided with instructions via email on how to download the My Grief app. To ensure engagement, a member of the research team contacted the families by phone to confirm the successful installment of the app 1 week after providing app access. The outcome variables were assessed at baseline (Time [T] 1, *N* = 126), as well as 3 months (T2, *n* = 67), 6 months (T3, *n* = 63), and 12 months (T4, *n* = 56) later. The study has received ethical approval from the Swedish Ethical Review Authority (No. 2021‐00770, No. 2021‐05333‐02) and has been carried out in accordance with The Code of Ethics of the World Medical Association (Declaration of Helsinki). All participants provided written informed consent.

#### My Grief app

For the 12‐month study duration, participants had access to the self‐management app My Grief, for ethical reasons. It should be noted that this deviates from the study protocol, which did not specify what would happen after the first 3 months of app access (Eklund et al., 2021). The app included (a) psychoeducation about grief and prolonged grief; (b) a self‐monitor function to track one's grief; (c) self‐guided exercises, such as mindfulness and writing exercises (e.g., exposure to memories of the loss); and (d) a support section (i.e., contact details for grief support, tips on how to expand one's social network, and recommendations for podcast and literature about grief). Users could self‐select which components and exercises they used. Participants self‐reported that in the first 3 months, they had used the psychoeducation (79%) and self‐monitoring sections (79%) most, followed by the self‐guided exercise section (64%) and support section (33%; for more information on My Grief app content and participant experiences see Eklund et al., 2021, 2022, 2024).

### Measures

#### Sociodemographic and loss‐related variables

At baseline, sociodemographic (i.e., gender, age, marital status, educational attainment, employment, country of birth) and loss‐related variables (i.e., deceased child's gender, age at death, cause of death, and diagnosis if they died due to illness) were assessed using a self‐constructed questionnaire.

#### App usage

At the 3‐, 6‐, and 12‐month follow‐up assessments, participants were asked to indicate their average app usage frequency since the last assessment point. Responses were rated on a 5‐point scale with response options of 1 (*daily*), 2 (*a few times a week*), 3 (*once a week*), 4 (*less than once a week*), or 5 (*I did not use the app*).

#### Prolonged grief symptoms

The PG‐13 (Prigerson et al., 2009; Swedish version: Pohlkamp et al., 2018) was used to assess prolonged grief symptoms. The PG‐13 comprises 13 items, including 11 items to assess cognitive, behavioral, and emotional symptoms. Participants rated the frequency of past‐month symptoms on a Likert scale ranging from 1 (*not at all*) to 5 (*several times a day/overwhelmingly*). PG‐13 scoring entails summing the 11 symptom items, with higher scores indicating higher symptom levels. The PG‐13 has demonstrated satisfactory psychometric properties in a Swedish bereaved sample (Pohlkamp et al., 2018). In this sample, the PG‐13 showed good‐to‐excellent reliability across the four assessment points, T1: Cronbach's α = .88, T2: Cronbach's α = .89, T3: Cronbach's α = .92, T4: Cronbach's α = .88.

#### PTSS

The PTSD Checklist for *DSM‐5* (PCL‐5; Weathers et al., 2013) was used to assess bereavement‐related PTSS based on symptoms outlined in the *Diagnostic and Statistical Manual of Mental Disorders* (5th ed.; *DSM‐5*; American Psychiatric Association, 2013). This self‐report measure consists of 20 items that are rated on a 5‐point scale ranging from 0 (*not at all*) to 4 (*extremely*), with scores summed and higher scores indicating higher levels of PTSS severity. The Swedish version of the PCL‐5 has shown satisfactory psychometric properties (Sveen et al., 2016). In the present sample, the PCL‐5 demonstrated excellent internal consistency at baseline, Cronbach's α = .92, and all follow‐up assessments, T2: Cronbach's α = .93, T3: Cronbach's α = .95, T4: Cronbach's α = .94.

#### Depressive symptoms

Depressive symptoms were measured using the nine‐item Patient Health Questionnaire (PHQ‐9, Kroenke et al., 2001), which assesses symptoms based on the *DSM‐5*. Items were rated on a 4‐point scale ranging from 0 (*not at all*) to 3 (*nearly every day*), with scores summed and higher scores indicating higher levels of depressive symptom severity. The Swedish adaptation has shown satisfactory psychometric properties (Hansson et al., 2009). In this sample, the PHQ‐9 demonstrated good‐to‐excellent internal consistency across all time points, T1: Cronbach's α = .87, T2: Cronbach's α = .90, T3: Cronbach's α = .89, T4: Cronbach's α = .90.

#### Grief‐related avoidance behavior

The Depressive and Anxious Avoidance in Prolonged Grief Questionnaire (DAAPGQ; Boelen & van den Bout, 2010) was used to measure grief‐related avoidance behaviors. The nine‐item questionnaire can be divided into two subscales: Depressive Avoidance of Activities (five items) and Anxious Avoidance of Loss‐Related Cues (four items). All items are rated on an 8‐point scale ranging from 1 (*not at all true for me*) to 8 (*completely true to me*). Scores are summed, with higher scores indicating higher levels of grief‐related avoidance. In this study, a Swedish translation of the DAAPGQ (Sveen et al., 2024) was used. At T1, the Depressive Avoidance subscale demonstrated excellent reliability, Cronbach's α = .90, and the Anxious Avoidance subscale demonstrated acceptable reliability, Cronbach's α = .70.

#### Grief‐related rumination

The Utrecht Grief Rumination Scale (UGRS; Eisma et al., 2014; Swedish version: Sveen et al., 2019) was used to measure grief‐related rumination. The instrument comprises 15 items assessing the past‐month frequency of specific thoughts, with responses rated on a 5‐point scale ranging from 1 (*never*) to 5 (*very often*). Scores are summed, with higher scores indicating higher levels of grief‐related rumination. The Swedish version demonstrated satisfactory psychometric properties in a sample of bereaved Swedish parents (Sveen et al., 2019). In this study, the UGRS showed excellent reliability at T1, Cronbach's α = .91.

#### Negative grief‐related cognitions

The Grief Cognition Questionnaire–Short Form (GCQ‐SF; Boelen & Lensvelt‐Mulders, 2005; Doering et al., 2020) was used to measure negative grief‐related cognitions. The measure comprises 18 items assessing negative cognitions about oneself, the world, and the future, as well as threatening misinterpretations of grief reactions. Items are rated on a 6‐point scale ranging from 0 (*strongly disagree*) to 5 (*strongly agree*). A Swedish translation of the GCQ‐SF was developed through a formal translation/back‐translation procedure. In this sample, the scale showed excellent reliability at T1, Cronbach's α = .92.

### Data analysis

Analyses were executed with IBM SPSS Statistics (Version 29). First, dropout analyses were conducted by comparing participants who filled out symptom measures at T4 (i.e., completers; *n* = 56) versus those who did not (i.e., dropouts; *n* = 70) on T1 variables. For categorical variables (i.e., gender, country of birth, educational attainment, employment, marital status, whether the respondent was living with the deceased child's other parent, number of children [including the deceased child], loss of another child, deceased child's gender, child's cause of death), chi‐square tests, or Fisher's exact tests if cell counts were less than five, were performed to compare dropouts to completers. To assess differences in parent age, age of the deceased child, and time since loss, as well as scores on main study variables (i.e., prolonged grief symptoms, PTSS, depressive symptoms, depressive and anxious avoidance, grief‐related rumination, and negative grief‐related cognitions) between dropouts and completers, independent samples *t* tests were conducted. Assumptions of homogeneity and normality were checked, and nonparametric alternative analyses (i.e., Mann–Whitney *U*‐test) were performed when these assumptions were violated.

To assess symptom change during continued access to the My Grief app, three matched–paired sample *t* tests for each outcome variable (i.e., prolonged grief symptoms, PTSS, and depressive symptoms) were conducted, comparing each follow‐up against the baseline (T1 vs. T2, T1 vs. T3, and T1 vs. T4). If homogeneity or normality assumptions were violated, a Wilcoxon signed‐rank test was used. Additionally, within‐group Cohen's *d* was calculated by subtracting the mean score of the dependent variable at T2, T3, and T4 from the mean score at T1, then dividing the result by the pooled standard deviation for both groups.

Further, nine multiple linear regression analyses were conducted to explore predictors of change in prolonged grief, posttraumatic stress, and depressive symptoms (assessed as absolute change scores for T1–T2, T1–T3, and T1–T4). In each regression analysis, T1 symptom levels, sample characteristics (i.e., time since death, child age, cause of death, educational attainment, gender, and app usage), and cognitive behavioral variables (i.e., depressive and anxious avoidance, grief‐related rumination, and negative grief‐related cognitions) were entered as independent variables simultaneously.

## RESULTS

### Sample characteristics

A total of 126 participants who received app access for 12 months were included. Most parents were female (80.2%). The mean parent age was 46.26 years (*SD* = 10.47), and most parents were born in Sweden (90.5%), had a university‐ or college‐level education (61.9%), were a student or employed (77.0%), and had a partner (89.7%). The loss of a child had occurred an average of 4.88 years (*SD* = 2.60) before baseline, and children were, on average, 10.38 years old (*SD* = 9.90) at the time of death. The most common cause of death was cancer (52.4%). For more details on the sample characteristics, see Table 1.

**TABLE 1:** Baseline sample characteristics

Characteristic	*n*	%	*M*	*SD*
Gender				
Female	101	80.2		
Male	25	19.8		
Age (years)			46.26	10.47
Country of birth				
Sweden	114	90.5		
Other	12	9.5		
Educational attainment				
University/college	78	61.9		
Lower/secondary school	48	38.1		
Employment status				
Employed/student	97	77.0		
Other^a^	29	23.0		
Marital status				
Together with partner	113	89.7		
Single or widow/widower	13	10.3		
Living with the other parent				
Yes	82	65.1		
No	44	34.9		
No. of children^b^				
1	11	8.7		
≥ 2	115	91.3		
Lost another child				
Yes	4	3.2		
No	115	91.3		
Data missing	7	5.6		
Child gender				
Female	44	34.9		
Male	79	62.7		
Nonbinary or missing data	3	2.4		
Age of deceased child			10.38	9.90
Cause of death				
Cancer	66	52.4		
Stillbirth	18	14.3		
Infant death (< 1 year)	11	8.7		
Accident	10	7.9		
Mental health problems	18	14.2		
Other causes	2	1.6		
Unknown	1	0.8		
Time since loss (years)			4.88	2.60

*Note*: *N* = 126.

^a^ Parental leave, sick leave, retirement leave, and “other.”

^b^ Including children who died and those over 18 years old.

### App usage

App usage was highest in the first 3 months of the study period (i.e., 96.7% of participants used the app between T1 and T2), and subsequently decreased from 3–6 months (30.2% between T2 and T3) and from 6–12 months (26.3% between T3–T4). From T1 to T2, most participants used the app less than once a week (52.2%), once a week (14.5%), or a few times per week (27.5%). As app usage strongly decreased over time, we solely used the frequency of app usage between T1 and T2 as a predictor of symptom change in our main analyses.

### Dropout analyses

Participants who started the T4 survey (*n* = 56) were compared to participants who did not (*n* = 70). No significant group differences emerged on sociodemographic and loss‐related variables, cognitive behavioral variables, and symptom levels. Dropout analyses are shown in Supplementary Table S1.

### Main analyses

#### Changes in prolonged grief symptoms, PTSS, and depressive symptoms

See Table 2 for means, standard deviations, and medians for all symptom measures at all time points. Baseline symptom scores for all participants who completed each follow‐up assessment are presented in Table 3. Significant decreases in prolonged grief symptoms were detected from T1 to T2, *t*(66) = 4.02, *p* < .001, *d* = 0.49; T1 to T3, *t*(62) = 4.31, *p* < .001, *d* = 0.54; and T1 to T4 (*n* = 56), *z* = −3.73, *p* < .001, *d* = 0.57. Two participants reported nonclinical levels of prolonged grief symptoms (i.e., PG‐13 scores less than 17) at T2 (3.0%), and five participants reported nonclinical levels at T3 (7.9%) and T4 (8.9%).

**TABLE 2:** Means, medians, and standard deviation for prolonged grief, posttraumatic stress, and depressive symptoms at Time (T) 1, T2, T3, and T4

	T1			T2			T3			T4		
Variable	(baseline)			(3‐month follow‐up)			(6‐month follow‐up)			(12‐month follow‐up)		
	*M*	*SD*	*Mdn*	*M*	*SD*	*Mdn*	*M*	*SD*	*Mdn*	*M*	*SD*	*Mdn*
Prolonged grief symptoms	33.16	8.86	34.00	29.90	9.33	28.00	30.37	10.24	30.00	29.23	9.31	27.50
Posttraumatic stress symptoms	26.89	14.93	25.50	20.20	13.92	17.00	24.05	18.04	22.00	20.89	15.24	18.00
Depressive symptoms	8.92	5.67	8.00	7.33	5.77	7.00	8.24	6.29	8.00	8.33	6.07	8.00

**TABLE 3:** Mean baseline symptoms among participants who completed each assessment

	T1 symptom scores	
Variable	*M*	*SD*
Prolonged grief symptoms		
T2 completers	33.24	8.25
T3 completers	34.10	8.67
T4 completers	33.77	7.95
Posttraumatic stress symptoms		
T2 completers	26.26	14.58
T3 completers	28.45	14.60
T4 completers	28.56	14.84
Depressive symptoms		
T2 completers	8.60	5.56
T3 completers	9.41	5.52
T4 completers	9.07	5.54

*Note*: T = Time.

Similarly, significant decreases in PTSS were detected from T1 to T2, *t*(65) = 4.78, *p* < 001, *d* = 0.59; T1 to T3 (*n* = 62), *z* = −3.13, *p* = .002, *d* = 0.30; and T1 to T4, *t*(54) = 4.88, *p* < .001, *d* = 0.66. Furthermore, significant decreases in depressive symptoms were detected from T1 to T2 (*n* = 67), *z* = −2.65, *p* = .008, *d* = 0.29, and from T1 to T3, *t*(62) = 2.09, *p* = .041, *d* = 0.26. No significant differences in depressive symptoms were detected between T1 and T4 (*n* = 55), *z* = −1.62, *p* = .106, *d* = 0.16.

#### Predictors of change in prolonged grief symptoms, PTSS, and depressive symptoms

Three multiple regressions were performed to examine predictors of change in prolonged grief symptoms from T1 to T2, T3, and T4 (Table 4). The model predicting T1–T2 prolonged grief symptom change was significant, *F*(11, 55) = 2.72, *p* = .007, *R* ^2^ = .35. Higher T1 prolonged grief symptoms, lower T1 negative grief‐related cognitions, the death of a younger child, and being a female (vs. male) parent were related to larger symptom reductions. The model predicting T1–T3 prolonged grief symptom change was not significant, *F*(11, 42) = 1.81, *p* = .083, *R* ^2^ = .32, nor was the model predicting T1–T4 prolonged grief symptom change, *F*(11, 37) = 1.53, *p* = .164, *R* ^2^ = .31.

**TABLE 4:** Multiple regression of predictors of prolonged grief symptom reductions

	T1–T2		T1–T3		T1–T4	
Dependent variable	*B*	95% CI	*B*	95% CI	*B*	95% CI
T1 prolonged grief symptoms	0.49	[0.20, 0.77]	0.58	[0.23, 0.94]	0.67	[0.25, 1.10]
T1 depressive avoidance	0.05	[−0.18, 0.28]	−0.22	[−0.51, 0.06]	−0.10	[−0.44, 0.24]
T1 anxious avoidance	−0.22	[−0.48, 0.04]	−0.05	[−0.37, 0.28]	−0.19	[−0.57, 0.20]
T1 grief‐related rumination	−0.03	[−0.20, 0.14]	−0.03	[−0.24, 0.19]	−0.06	[−0.32, 0.20]
T1 negative grief‐related cognitions	−0.15^*^	[−0.28, ‐0.03]	−0.21^*^	[−0.37, ‐0.04]	−0.15	[−0.34, 0.05]
Time since loss	−0.14	[−0.77, 0.50]	−0.09	[−0.89, 0.70]	0.13	[−0.81, 1.07]
Child age	−0.26	[−0.44, ‐0.09]	−0.15	[−0.37, 0.07]	−0.19	[−0.45, 0.08]
Cause of death	1.91	[−1.55, 5.37]	0.94	[−3.39, 5.26]	1.17	[−3.97, 6.31]
Educational attainment	0.66	[− 2.62, 3.94]	0.43	[−3.67, 4.53]	−2.17	[−7.04, 2.70]
Gender	4.43^*^	[0.46, 8.41]	−0.27	[−5.24, 4.70]	3.98	[−1.92, 9.88]
App usage	2.91	[−0.24, 6.06]	1.49	[−2.44, 5.43]	3.82	[−0.86, 8.49]
Model statistics	*F* = 2.72, *R* ^2^ = .35		*F* = 1.81, *R* ^2^ = .32		*F* = 1.53, *R* ^2^ = .31	

*Note*: Dummy coding was used for cause of death (cancer = 1, other = 0), educational attainment (university and college = 1, other = 0), gender (female = 1, male = 0), and app usage (daily, a few times a week, once a week = 1, other = 0). T = Time; CI = confidence interval.

* *p* < .05. **p < .01.

Three multiple regressions were performed to examine predictors of change in PTSS in T2, T3, and T4 (Table 5). The model predicting T1–T2 PTSS change was significant, *F*(11, 54) = 3.32, *p* = .002, *R* ^2^ = .40. Higher T1 PTSS, lower T1 negative grief‐related cognitions, and younger age of the child at death were related to larger reductions in PTSS. The model predicting T1–T3 PTSS was not significant, *F*(11, 41) = 1.19, *p* = .327, *R* ^2^ = .24, nor was the model predicting T1–T4 PTSS, *F*(11, 36) = 1.43, *p* = .203, *R* ^2^ = .30.

**TABLE 5:** Multiple regression of predictors of posttraumatic stress symptom reductions

	T1–T2		T1–T3		T1–T4	
Dependent variable	*B*	95% CI	*B*	95% CI	*B*	95% CI
T1 posttraumatic stress symptoms	0.48^**^	[0.22, 0.73]	0.55^*^	[0.08, 1.03]	0.35	[−0.04, 0.74]
T1 depressive avoidance	0.09	[−0.24, 0.42]	−0.14	[−0.75, 0.48]	0.08	[−0.59, 0.42]
T1 anxious avoidance	−0.32	[−0.71, 0.06]	−0.07	[−0.78, 0.64]	−0.17	[−0.76, 0.41]
T1 grief‐related rumination	0.12	[−0.14, 0.38]	0.04	[−0.44, 0.52]	0.27	[−0.12, 0.66]
T1 negative grief‐related cognitions	−0.23^*^	[−0.42, 0.03]	−0.36	[−0.72, 0.01]	−0.20	[−0.50, 0.09]
Time since loss	0.06	[−0.85, 0.97]	−0.84	[−2.51, 0.83]	0.21	[−1.17, 1.58]
Child age	−0.41^**^	[−0.67, ‐0.16]	−0.23	[−0.70, 0.23]	0.03	[−0.35, 0.41]
Cause of death	5.04	[0.01, 10.07]	4.93	[−4.31, 14.18]	8.32^*^	[0.72, 15.93]
Educational attainment	0.11	[−4.69, 4.91]	−5.21	[−14.03, 3.61]	−2.60	[−9.85, 4.66]
Gender	4.83	[−1.02, 10.69]	6.75	[−4.02, 17.52]	2.32	[−6.54, 11.18]
App usage	1.07	[−3.55, 5.70]	4.08	[−4.42, 12.08]	3.72	[−3.28, 10.71]
Model statistics	*F* = 3.32^**^, *R* ^2^ = .40		*F* = 1.19, *R* ^2^ = .24		*F* = 1.43, *R* ^2^ = .30	

*Note*: Dummy coding was used for cause of death (cancer = 1, other = 0), educational attainment (university and college = 1, other = 0), gender (female = 1, male = 0), and app usage (daily, a few times a week, once a week = 1, other = 0). T = Time; CI = confidence interval.

* *p* < .05. **p < .01.

Three multiple regressions were performed to examine predictors of change in depressive symptoms from T1 to T2, T3, and T4 (Table 6). The model predicting T1–T2 depressive symptom change was significant, *F*(11, 55) = 2.31, *p* = .021, *R* ^2^ = .32. Higher T1 depressive symptoms and lower T1 anxious avoidance were related to larger reductions in depressive symptoms. The model predicting T1–T3 depressive symptom change was also significant, *F*(11, 42) = 2.14, *p* = .038, *R* ^2^ = .36. Higher T1 depressive symptoms were related to larger reductions in T3 depressive symptoms. The model predicting T1–T4 depressive symptom change was not significant, *F*(11, 36) = .72, *p* = .714, *R* ^2^ = .18.

**TABLE 6:** Multiple regression of predictors of depressive symptom reductions

	T1–T2		T1–T3		T1–T4	
Dependent variable	*B*	95% CI	*B*	95% CI	*B*	95% CI
T1 depressive symptoms	0.37	[0.11, 0.63]	0.54	[0.25, 0.83]	0.20	[−0.18, 0.58]
T1 depressive avoidance	0.04	[−0.11, 0.20]	−0.13	[−0.30, 0.05]	−0.04	[−0.27, 0.19]
T1 anxious avoidance	−0.23^*^	[−0.40, ‐0.05]	−0.14	[−0.33, 0.06]>	−0.16	[−0.42, 0.09]
T1 grief‐related rumination	−0.02	[−0.14, 0.09]	−0.04	[−0.16, 0.09]	0.04	[−0.13, 0.20]
T1 negative grief‐related cognitions	−0.02	[−0.11, 0.06]	−0.08	[−0.18, 0.01]	0.00	[−0.12, 0.13]
Time since loss	0.20	[−0.22, 0.61]	0.03	[−0.44, 0.50]	−0.03	[−0.64, 0.58]
Child age	0.09	[−0.20, 0.03]	−0.04	[−0.17, 0.9]	0.07	[−0.10, 0.23]
Cause of death	1.19	[−1.09, 3.47]	1.25	[−1.31, 3.81]	1.69	[−1.66, 5.04]
Educational attainment	0.19	[−2.01, 2.38]	−0.19	[−2.66, 2.27]	−1.09	[−4.30, 2.13]
Gender	2.64	[−0.03, 5.31]	1.95	[−1.05, 4.94]	0.78	[−3.14, 4.69]
App usage	1.58	[−0.57, 3.73]	0.76	[−1.66, 3.17]	1.08	[−2.07, 4.23]
Model statistics	*F* = 2.31^*^, *R* ^2^ = .32		*F* = 2.14^*^, *R* ^2^ = .36		*F* = 0.72, *R* ^2^ = .18	

*Note*: Dummy coding was used for cause of death (cancer = 1, other = 0), educational attainment (university and college = 1, other = 0), gender (female = 1, male = 0), and app usage (daily, a few times a week, once a week = 1, other = 0). T = Time; CI = confidence interval.

* *p* < .05. **p < .01.

## DISCUSSION

The primary aim of the present study was to shed light on changes in postloss symptomatology during continued access to the My Grief app. In line with our predictions, small‐to‐moderate reductions in prolonged grief symptoms and PTSS were observed at 3, 6, and 12‐month follow‐up compared to baseline. Although small reductions in depressive symptoms were also observed at 3‐ and 6‐month follow‐ups relative to baseline, no statistically significant differences emerged from baseline to 12‐month follow‐up. The maintenance of the effects on most mental health indicators among participants with app access aligns with prior research on guided internet‐based therapies showing the maintenance of short‐term effects on symptoms of prolonged grief, posttraumatic stress, and depression over 1–1.5 years (Kersting et al., 2013; Wagner & Maercker, 2007). The inconsistent effects on depressive symptoms complement results from between‐group comparisons in our RCT (Sveen et al., 2024), demonstrating that app access did not significantly change depressive symptoms over 3 months in the intervention arm relative to a waitlist‐control group. Generally, the findings align with results from other internet‐based grief interventions showing larger effects on prolonged grief symptoms and PTSS than depressive symptoms (Wagner et al., 2020).

The secondary aim of the study was to explore predictors of symptom change following app access. The most consistent predictor of larger prolonged grief, posttraumatic stress, and depressive symptom reductions was higher baseline symptom severity. This aligns with findings from earlier studies examining predictors of face‐to‐face CBT (Boelen et al., 2011) and therapist‐guided internet‐based therapy for prolonged grief (Schmidt et al., 2022) in which baseline prolonged grief symptom severity emerged as a significant predictor of pre‐to posttreatment prolonged grief symptom reductions. These findings complement the well‐established finding that psychological treatment for bereaved individuals is effective among people with elevated symptom levels, whereas universal interventions for all bereaved adults are not effective (e.g., Currier et al., 2008). We tentatively conclude that people with more severe psychopathology also benefit more from guided and unguided internet‐based grief interventions.

Following access to the My Grief app, 3‐month symptom changes were predicted by some baseline cognitive behavioral variables. Participants who endorsed more negative grief‐related beliefs about the self, the world, and the future, as well as catastrophic misinterpretations of grief reactions, showed smaller reductions in prolonged grief symptoms and PTSS at 3‐month follow‐up. Participants with higher anxious avoidance (i.e., cognitive and behavioral avoidance of reminders of the loss) showed smaller reductions in depressive symptoms at 3‐month follow‐up. People with more severe avoidance and related negative cognitions may benefit less from unguided therapeutic exercises. For example, writing exercises on memories of the death, made available in the My Grief app environment and designed to reduce loss‐related avoidance and associated catastrophic misinterpretations of grief reactions, may require therapist guidance to be optimally effective (Sveen et al., 2024; van der Houwen et al., 2010). However, our findings also echo results from trials of cognitive behavioral therapies showing that people with higher trauma‐related avoidance and negative grief cognitions demonstrated smaller prolonged grief symptom reductions (Bryant et al., 2017; Skritskaya et al., 2020). An alternative explanation may, therefore, be that people with more severe avoidance tendencies and negative cognitions have more difficulty engaging in or benefiting from interventions that promote confrontation with the irreversibility, implications, and emotions related to the loss. Developing and testing interventions to improve the acceptability and engagement with such cognitive behavioral techniques appears to be an important direction for future research.

Some sociodemographic and loss‐related variables also predicted symptom change. Female participants showed stronger prolonged grief symptom reductions than male participants at 3‐month follow‐up. It is possible that female participants find it easier to engage with exercises focused on monitoring and managing negative loss‐related emotions, as they have a stronger need to express feelings (Stroebe et al., 2001). The death of a younger child was related to larger reductions in prolonged grief symptoms and PTSS from baseline to 3‐month follow‐up. It is difficult to say what may have caused this finding. Perhaps, some exposure exercises were more efficacious if a child was younger at death, as it is potentially a more stressful life event for which emotional processing might be more effective. A notable null result was the nonsignificant effect of app usage on symptom change. This could imply that merely having access to an app is helpful to bereaved persons because it makes them feel supported. Alternatively, the association between app use and symptom change could be nonlinear, for example because using some components only a few times (e.g. self‐monitoring; Eisma et al., 2024) cause the effects of the app or because app‐induced reductions in mental health problems can cause less app usage (Eklund et al., 2024).

Some limitations of the current study warrant mention. First, due to study dropout, we had a relatively small sample at the 6‐ and 12‐month follow‐ups, which may have reduced our ability to detect long‐term reductions in depressive symptoms and, particularly, predictors of long‐term symptom reductions. Second, the sample predominantly consisted of female parents with higher levels of educational attainment, and all participants had experienced the death of a child. Future research should establish whether the findings related to long‐term effects and predictors of symptom change generalize to other subpopulations of bereaved adults. Third, due to ethical reasons, participants in the control group received access to the My Grief app after 3 months (Sveen et al., 2024). Therefore, we could not compare estimates of long‐term effects against a control group. Fourth, in this study, we gathered self‐reported data on app use (for a comprehensive discussion, see Eklund et al., 2024), which is subject to recall biases. Therefore, we advise collecting objective app usage data (e.g., the number and duration of interactions with features of the app, time per session) in future studies to enable digital phenotyping (i.e., a combination of objective and subjective usage data; Davidson, 2022) and, thereby, a more fine‐grained analysis of predictors of the effects of app access, including the elucidation of linear and nonlinear dose–response relationships.

Despite these limitations, our findings suggest that the beneficial effects of the My Grief app on prolonged grief symptoms and PTSS are maintained for up to 1 year following app access. Moreover, the results suggest that people with more severe loss‐related psychopathology may derive the most benefit from app access, whereas those with more negative grief‐related cognitions and loss‐related avoidance may derive less. Together with the findings from the controlled analyses in a previous RCT (Sveen et al., 2024), our results illustrate the potential applicability of the My Grief app as a complementary tool to existing therapist‐guided grief interventions.

## OPEN PRACTICES STATEMENT

The randomized controlled trial, which we report on in this article, was preregistered (Clinicaltrials.gov: NCT04552717). The analyses presented in this paper were not specifically preregistered. Neither the data nor the materials have been made available in a permanent third‐party archive. Requests for the data, syntax, output and materials can be sent via email to the corresponding author at m.c.eisma@rug.nl.

## AUTHOR NOTE

This work was supported by the Swedish Childhood Cancer Fund (PR2018‐0047; TJ2018‐0002) and the Swedish Infant Death Foundation.

The funder did not play a role in the study design; the collection, analysis, or interpretation of the data; the writing of the report; or the decision to submit the article for publication.

The authors wish to extend special thanks to the parents who participated in this study using the app. We would also like to thank the National Center for PTSD, the U.S. Department of Defense, and the Defense Health Agency Connected Health branch for granting permission to base the My Grief app on the source code of the PTSD Coach.

## Supporting information




**Supplemental Table A** Comparisons of Characteristics of Completers and Dropouts at T1
