# Supplementary material for: A 1‐year follow‐up of the My Grief app for prolonged grief
Source: J Trauma Stress. 2025 Jun 20;38(6):952–62. doi: 10.1002/jts.23181 (PMC12711450; doi:10.1002/jts.23181)
Supplement: Supplementary file 1 — Supplemental Table A Comparisons of Characteristics of Completers and Dropouts at T1 [file JTS-38-952-s001.docx]

**Supplemental Table A**

*Comparisons of Characteristics of Completers and Dropouts at T1*

| Characteristics | | Completers  *n* = 56 | Dropouts  *n* = 70 | Group Comparisons |
| --- | --- | --- | --- | --- |
| Gender, *n* (%) | |  |  |  |
|  | Female | 49 (87.5) | 52 (74.3) | χ^2^ (1, *N* = 126) = 3.416, *p* = .065 |
|  | Male | 7 (12.5) | 18 (25.7) |  |
| Age, *M* (*SD*) | | 47.14 (10.43) | 45.55 (10.52) | *t* (124) = -.847, *p* = .399 |
| Country of birth, *n* (%) | |  |  |  |
|  | Sweden | 51 (91.1) | 63 (90.0) | χ^2^ (1, *N* = 126) = .041, *p* = .839 |
|  | Other | 5 (8.9) | 7 (10.0) |  |
| Educational level, *n* (%) | |  |  |  |
|  | University/College | 38 (67.9) | 40 (57.1) | χ^2^ (1, *N* = 126) = 1.514, *p* = .218 |
|  | Lower/Secondary School | 18 (32.1) | 30 (42.9) |  |
| Employment, *n* (%) | |  |  |  |
|  | Employed/Student | 44 (78.6) | 53 (75.7) | χ^2^ (1, *N* = 126) = .143, *p* = .705 |
|  | Other^a^ | 12 (21.4) | 17 (24.3) |  |
| Marital status, *n* (%) | |  |  |  |
|  | Married, cohabiting, together | 48 (85.7) | 65 (92.9) | χ^2^ (1, *N* = 126) = 1.715, *p* = .190 |
|  | Single or Widowed | 8 (14.3) | 5 (7.1) |  |
| Living with the other parent, *n* (%) | |  |  |  |
|  | Yes | 38 (67.9) | 44 (62.9) | χ^2^ (1, *N* = 126) = 0.342, *p* = .559 |
|  | No | 18 (32.1) | 26 (37.1) |  |
| No. of children^b, c^, *n* (%) | |  |  |  |
|  | 1 | 7 (12.5) | 4 (5.7) | *p* = .215 |
|  | 2+ | 49 (87.5) | 66 (94.3) |  |
| Lost another child^c^, *n* (Valid %) | |  |  |  |
|  | Yes | 0 (0) | 4 (5.8) | *p* = .138 |
|  | No | 50 (100) | 65 (94.2) |  |
| Child Gender, *n* (%) | |  |  |  |
|  | Male | 34 (60.7) | 45 (64.3) | χ^2^ (1, *N* = 126) = 0.170, *p* = .680 |
|  | Female, Non-binary and Not reported | 22 (39.3) | 25 (35.7) |  |
| Age of deceased child, *M* (*SD*) | | 10.53 (10.10) | 10.26 (9.80) | 𝑈 = 1961.5, *p* = .994 |
| Cause of death, *n* (%) | |  |  |  |
|  | Cancer | 24 (42.9) | 42 (60.0) | χ^2^ (1, *N* = 126) = 3.665, *p* = .056 |
|  | Other causes^d^ | 32 (57.1) | 28 (40.0) |  |
| Time since loss (in years), *M* (*SD*) | | 5.13 (2.54) | 4.68 (2.64) | 𝑈 = 1699, *p* = .200 |
| Prolonged grief symptoms, *M* (*SD*) | | 33.77 (7.95) | 32.67 (9.56) | 𝑈 = 1839.5, *p* = .554 |
| Posttraumatic stress symptoms, *M* (*SD*) | | 28.55 (14.71) | 25.56 (15.07) | 𝑈 = 1713, *p* = .225 |
| Depressive symptoms, *M* (*SD*) | | 9.05 (5.29) | 8.81 (6.00) | 𝑈 = 1907, *p* = .794 |
| Grief-related depressive avoidance behavior, *M* (*SD*) | | 21.93 (8.11) | 21.56 (10.07) | 𝑈 = 1863.5, *p* = .635 |
| Grief-related anxious avoidance behavior, *M* (*SD*) | | 13.96 (6.75) | 13.87 (6.47) | 𝑈 = 1973.5, *p* = .947 |
| Grief-related rumination, *M* (*SD*) | | 48.89 (14.28) | 46.53 (13.10) | *t* (124) = –.968, *p* = .335 |
| Grief-related negative cognitions, *M* (*SD*) | | 31.52 (16.98) | 29.47 (20.88) | 𝑈 = 1747.5, *p* = .297 |

*Note*. ^a^ Parental leave, sick leave, retirement leave, and other ^b^ Including those who died and children over 18 years ^c^ Fisher's exact test was used due to cell counts < 5, data from 7 cases was missing; ^d^ Stillbirth, infant death (under 1 year), accident, mental health problems, other causes and unknown cause of death; Completers = participants who started filling out the T4 survey. Dropouts = those who did not start filling out the T4 survey.
